# ICOSeg: Real-Time ICOS Protein Expression Segmentation from Immunohistochemistry Slides Using a Lightweight Conv-Transformer Network

**DOI:** 10.3390/cancers14163910

**Published:** 2022-08-13

**Authors:** Vivek Kumar Singh, Md. Mostafa Kamal Sarker, Yasmine Makhlouf, Stephanie G. Craig, Matthew P. Humphries, Maurice B. Loughrey, Jacqueline A. James, Manuel Salto-Tellez, Paul O’Reilly, Perry Maxwell

**Affiliations:** 1 Precision Medicine Centre of Excellence, The Patrick G Johnston Centre for Cancer Research, Queen’s University Belfast, Belfast BT9 7AE, UK; 2 National Subsea Centre, Robert Gordon University, Aberdeen AB21 0BH, UK; 3 Leeds Teaching Hospitals NHS Trust, Leeds LS1 3EX, UK; 4 Leeds Institute of Cancer and Pathology, University of Leeds, St. James’ University Hospital, Leeds LS9 7TF, UK; 5 Cellular Pathology, Belfast Health and Social Care Trust, Belfast City Hospital, Lisburn Road, Belfast BT9 7AB, UK; 6 Regional Molecular Diagnostic Service, Belfast Health and Social Care Trust, Health Sciences Building, Queen’s University Belfast, Belfast BT9 7AE, UK; 7 Northern Ireland Biobank, The Patrick G Johnston Centre for Cancer Research, Queen’s University Belfast, Belfast BT9 7AE, UK; 8 Division of Molecular Pathology, The Institute of Cancer Research, Sutton SM2 5NG, UK; 9 Sonrai Analytics Ltd., Lisburn Road, Belfast BT9 7BL, UK

**Keywords:** colon cancer, immunohistochemistry, ICOS, deep learning, channel attention

## Abstract

**Simple Summary:**

Inducible T-cell COStimulator (ICOS) is a biomarker of interest in checkpoint inhibitor therapy, and as a means of assessing T-cell regulation as part of a complex process of adaptive immunity. The aim of our study is to segment the ICOS positive cells using a lightweight deep-learning segmentation network. We aim to assess the potential of a convolutional neural network and transformer together that permits the capture of relevant features from immunohistochemistry images. The proposed study achieved remarkable results compared to the existing biomedical segmentation methods on our in-house dataset and surpassed our previous analysis by only utilizing the Efficient-UNet network.

**Abstract:**

In this article, we propose ICOSeg, a lightweight deep learning model that accurately segments the immune-checkpoint biomarker, Inducible T-cell COStimulator (ICOS) protein in colon cancer from immunohistochemistry (IHC) slide patches. The proposed model relies on the MobileViT network that includes two main components: convolutional neural network (CNN) layers for extracting spatial features; and a transformer block for capturing a global feature representation from IHC patch images. The ICOSeg uses an encoder and decoder sub-network. The encoder extracts the positive cell’s salient features (i.e., shape, texture, intensity, and margin), and the decoder reconstructs important features into segmentation maps. To improve the model generalization capabilities, we adopted a channel attention mechanism that added to the bottleneck of the encoder layer. This approach highlighted the most relevant cell structures by discriminating between the targeted cell and background tissues. We performed extensive experiments on our in-house dataset. The experimental results confirm that the proposed model achieves more significant results against state-of-the-art methods, together with an 8× reduction in parameters.

## 1. Introduction

According to the World Health Organization (WHO), colorectal cancer (CRC) is the third most fatal cancer, causing approximately 0.9 million deaths each year. By the year 2030, the number of new cases is anticipated to rise to 2.2 million per year, resulting in 1.1 million fatalities worldwide [1]. An early diagnosis with proper screening could help to prevent colorectal cancer. Additionally, early identification may provide a better treatment response to prevent cancer cells spread and save lives.

The number of molecular stratified therapy opportunities targeting metastatic CRC is rising with the application of molecular biomarkers to benefit prognosis and treatment decision-making [2]. For instance, patients with metastatic microsatellite instability (MSI-H)/mismatch repair deficient (dMMR) tumors can benefit from immunotherapy, and MSI/MMR testing can be employed as a marker of genetic instability with therapeutic implications [3]. Other innovative therapeutic medicines are approved based on the results of a companion biomarker, such as PD-L1 immunohistochemistry (IHC) [4]. Translational oncology has been improved significantly by tissue-based biomarker analysis using IHC, which maintains spatial and cell-specific information, and allows for reliable biomarker expression analysis inside the tumor micro-environment [5]. A pathologist can assess the quantification of biomarker-expressing cells and their location to provide significant prognostic patient information [6,7]. As a means of evaluating biomarkers in a high volume/high throughput manner, Tissue microarrays (TMA) can be incorporated into the development pipeline of biomarker research [8,9]. Ultimately, manual TMA examination with IHC is a long and laborious procedure in evaluating biomarker-expressing cells, making it unsuitable for effective TMA investigation [10,11]. As a result, a Computer-Aided Diagnosis (CAD) system is necessary for examining the TMA and helping to train pathologists in their investigation workflows.

With today’s CAD image analysis technology, large-scale quantitative evaluation of IHC on TMAs is feasible. Recently, there has been a lot of research conducted on the advantages of computer-assisted quantitative cell count studies [12], cell segmentation tasks [13], and the effect of the automatic versus manual estimation [14]. Current artificial intelligence (AI) guided CAD systems show promising outcomes using deep learning algorithms in digital pathology, for example in predicting colon cancer metastases [15], nuclei segmentation [16], lymphocyte identification [17], in assessing tumor grade [18], and predicting molecular signatures [19]. Most of the developed CAD systems focus on identifying tiles in whole-slide images. These CAD systems have some limitations for successfully segmenting biomarker-expressing cells on IHC-stained tissue images. In our previous work [20], the evaluation of immune and immune-checkpoint biomarkers proposed utilizing deep learning techniques by automated identification of cells positive for the ICOS. The results were encouraging, but the Efficient-UNet model utilized a huge number of parameters (i.e., 66 million), making them difficult to implement in real-world clinical situations.

There is always a trade-off between the model complexity in terms of parameters and its performance. Usually, an attempt to reduce the number of trainable parameters through a pruning strategy or adopting a lightweight model may achieve the lower results. Therefore, it is important to design a model with lower computational complexity while keeping a similar performance or achieving better results. The lightweight model deployment creates many opportunities in various applications in pathology. It offers the use of a digital image without requiring additional processing resources, and allows to perform image analysis. Moreover, the application of this work will help to deploy on a cloud server where a pathologist can easily access to segment the positive cells from IHC slides.

**Figure 1:**
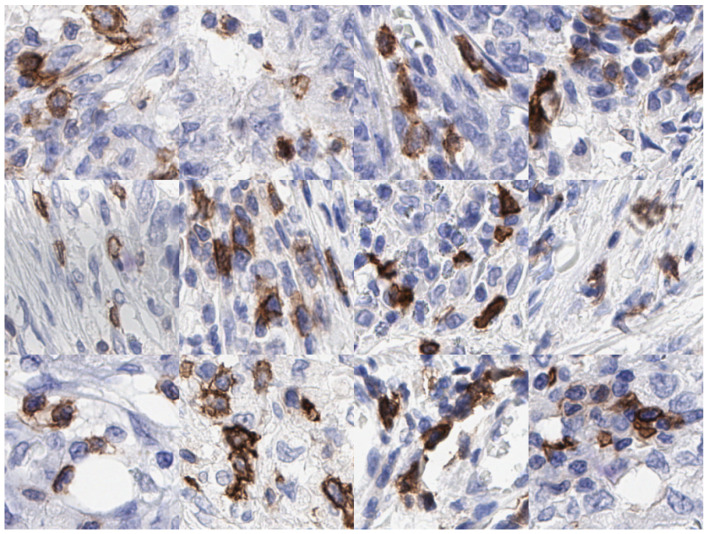
Examples of IHC patches extracted at 40× magnification containing ICOS positive (i.e., brown cytoplasmic and blue nuclear staining) and ICOS negative (blue nuclear stain only) cells in colon cancer.

We propose a lightweight model viz ICOSeg in segmenting ICOS positive cells. ICOS is a biomarker of interest in checkpoint inhibitor therapy and as a means of assessing T-cell regulation as part of a complex process of adaptive immunity. We merge the convolutional neural network (CNN) and transformer to capture the local and global feature representation with lower computational complexity. Figure 1 shows examples of patches, which highlight the ICOS positive cells in brown extracted from IHC slides. This study is an extension of our previous work [20] and shows higher computational complexity for similar tasks.

The main contributions of this paper are listed as follows:

- We propose an efficient, lightweight segmentation method called ICOSeg in segmenting ICOS positive cells that combines the CNN and transformer as a feature extractor with a lower latency rate. The proposed model extracts the local spatial features through CNN, and global representation learned using transformer block;
- We use the channel attention mechanism in the final encoder layer that enables the network to extract rich features and discriminate between the targeted cell’s structure and background pixels;
- We perform extensive experiments incorporating ablation studies and employ four pixel-wise evaluation metrics (i.e., Dice coefficient, aggregated Jaccard index, sensitivity, and specificity) that confirm the effectiveness of the proposed method;
- Experimental results confirm that ICOSeg efficiently outperforms several state-of-the-art methods (i.e., U-Net, Attention U-Net, FCN, DeepLabv3+, U-Net++, and Efficient U-Net) with lower trainable parameters.

The remainder of this paper is structured as follows. Section 2 discusses the proposed ICOSeg method in detail. The dataset description with experimental plans and limitations of our work is explained in Section 3. We conclude by summarizing our findings and suggest some future lines of research in Section 4.

## 2. Methods

In this section, we present the architectural details of the proposed ICOSeg segmentation model motivated by the existing work [21].

### 2.1. ICOSeg Architecture

**Figure 2:**
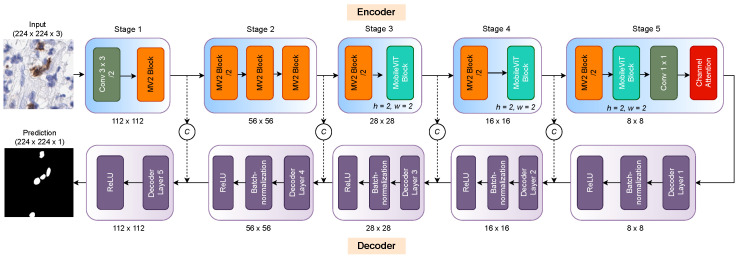
Overview of proposed segmentation model. The input IHC patch image feed to the encoder layer that extracts the spatial and global feature information. The decoder reconstructs the features into the segmentation map. Where *C* refers to the feature concatenation. The dash (- -) line corresponds to the skip connection between each encoder and decoder layers.

The proposed model consists of an encoder and decoder network. The IHC slide patch containing cells feeds to encoding layers, extracting the relevant spatial and global features. However, decoding layers allows reconstructing the feature representation into a segmentation map. We explore the encoder structure by [21], that merges the potential of learning local and global features of convolutions and transformers, respectively. Figure 2 shows the general overview of the proposed ICOSeg positive cell segmentation model.

**Encoder:** This consisted of five key stages using convolutional and MobileVit blocks. The first stage combined the two layers with a strided 3 × 3 convolutional layer that helped to extract the spatial features from the input patch images. The extracted feature maps are down-sampled by the factor of 2. MobileNetv2 (i.e., MV2) block [22] helped encode the cell’s feature maps in low-dimensional spaces and advanced feature representation power. Note that MV2 blocks in the encoder were mainly responsible for the down-sampling of the resolution of the intermediate feature maps. Stage two incorporated three MV2 blocks that allowed for enriching the spatial features and applied feature down-sampling. Stage three integrated the MV2 block and MobileViT block which, together, captured the global representation of the features. The MobileViT block learned contextual information with less computational complexity. In addition, it merged the advantage of Transformer and convolution. Stage four follows the stage three combination and featured maps downsampled by a factor of 2. The final stage combined the MV2 block, MobileViT block, and point-wise convolutional layers, which provided the bottleneck features that fed to the decoder layers. All the encoding layers helped to extract salient features, such as shape, texture, intensity, and margin. The last encoder layer used the channel attention [23] mechanism that highlights the targeted cell-related features and distinguished between relevant and the background pixels. It dynamically recalibrated channel-wise feature maps feedback through modeling precisely the interdependencies between channels.

The main functionality of a MobileViT block required an input tensor $I\;\in \;R^{h\times w\times }$ and involved an n × n convolutional layer followed by a point-wise convolution to create the map of $I_{L}\;\in \;R^{h\times w\times d}$. This convolutional layer enabled learning feature representations to capture local information, such as shape or intensity. To learn the global representation, unfolded $I_{L}$ into another *N* non-overlapped vector (i.e, patch) with a shape $I_{U}\;\in \;R^{p\times N_{patch}\times d}$. Where *p* is the area of a small patch and $N_{patch}\;=\;\frac{hw}{p}$ in which *h* and *w* refer to the height and width of that specific patch. To overcome the issue of not losing the spatial order of pixels, inter-patch connections are encoded using Transformers.

(1) $$I_{G}\left(p\right)\;=\;\;\mathrm{Transformer}\;\left(I_{U}\left(p\right)\right),\;1\;\leq \;p\;\leq \;P$$

where p ∈ {1…P}. Further, the output vector $I_{g}$ was again folded into a vector of shape to receive $I_{F}\;\in \;R^{h\times w\times d}$. This output feature vector used the point-wise (1 × 1) convolutional layer to project into a low dimensional space. This resultant feature was concatenated with the input feature map of *I*. To merge these concatenated features, an n × n convolutional layer was applied.

**Decoder:** We employed five decoding layers with a kernel size of 4 × 4 and stride of 2 × 2 that downsampled the features by two. Each decoder layer was followed by batch normalization and the non-linear ReLU activation function helped to confound the overfitting. The skip connection was used in between the encoding and decoding layers to narrow down the semantic gaps during feature reconstruction at the decoding stage. We used a threshold value of 0.5 to generate the binary segmentation map.

### 2.2. Loss Function

In this study, we used the combination of Binary cross-entropy (BCE) and Dice loss functions. The BCE loss can be formulated as follows:

(2) $$L^{\mathrm{BCE}}\left(gt_{mask},\;p_{mask}\right)\;=\;-(gt_{mask}\cdot log\left(p_{mask}\right)\;+\;\left(1\;-\;gt_{mask}\right)\cdot log\left(1\;-\;p_{mask}\right)),$$

Additionally, the Dice loss can be explained as follows:

(3) $$L^{\mathrm{Dice}}\left(gt_{mask},\;p_{mask}\right)\;=\;1\;-\;Dice\left(gt_{mask},\;p_{mask}\right)\;=\;1\;-\;\frac{2|gt_{mask}\left|\cdot \right|p_{mask}|}{|gt_{mask}{|}^{2}\;+\;{\left|p_{mask}\right|}^{2}},$$

Therefore, the total loss is the weighted summation of $L^{\mathrm{BCE}}$ and $L^{\mathrm{Dice}}$ described below:

(4) $$L^{\mathrm{Total}}(gt_{mask},\;p_{mask})\;=\;\gamma L^{\mathrm{BCE}}\left(gt_{mask},\;p_{mask}\right)\;+\;\left(1\;-\;\gamma \right)L^{\mathrm{Dice}}\left(gt_{mask},\;p_{mask}\right)$$

where $gt_{mask}$ is the ground truth mask, $p_{mask}$ corresponds to predicted mask generated by the model, and γ is weighting factor. Here, we used the γ equal to 0.4.

## 3. Experimental Results and Discussion

### 3.1. Dataset

In this article, we used the ICOS IHC data from our previous research [20]. ICOS-stained IHC slides were scanned with 40× obj. magnification. These are relatively large in pixels fed into the input of deep models. Therefore, we generated definitive input patches that were employed to train models. The patches were 256 × 256 pixels in size. The entire dataset was split into training, validation, and test sets with the ratio of 60%, 10%, and 30%, respectively, which is the same split, that was used in the previous work [20].

### 3.2. Training Details

We used the input patch and rescaled with the size of 224 × 224 pixels. The extracted patches were normalized from 0–255 pixel values to the range 0–1. An ADAM optimizer with an initial learning rate of 0.0001 was employed to optimize the proposed model reasonably. During the training, we applied the step decay if the dice coefficient on a validation set did not increase two consecutive epochs. We used a batch size of 8 patch images and trained the model from scratch for 50 epochs. To increase the samples size, we performed the data augmentation approach consisting of rotation at 90 degrees, horizontal flipping, and scaling with a probability of 0.5. The selected strategy was used to mitigate overfitting and provide more variability of features in the training network.

**Model development platform:** We developed the model using the PyTorch neural network library on the National High-Performance Computing (HPC) Kelvin-2 managed by Queen’s University Belfast (QUB). The model was trained on the 32 GB GPU memory with CUDA version 11.2. We evaluated the model on our local workstation, including GPU RTX2080 Ti of 11 GB video RAM. It is worthwhile to note that all the computational measurement and frame per second (FPS) speed was computed on a local workstation.

### 3.3. Evaluation Metrics

We pixel-wise assessed each method’s segmentation results using four evaluation metrics. It includes the dice coefficient score (Dice), Aggregated Jaccard Index (AJI), sensitivity, and specificity. These metrics were measured using true positive (TP), false positive (FP), false-negative (FN) rates, and true negative (TN). We assumed $gt_{mask}$ and $p_{mask}$ are the ground truth mask and predicted mask generated by the model for the same input patch images, respectively. The common area of segmented cells in $gt_{mask}$ and $p_{mask}$, referred to $TP\;=\;gt_{mask}\;\cap \;p_{mask}$. The segmented region which is out of the common region to $gt_{mask}$ corresponds to the FP that is defined as $\bar{gt}{}_{mask}\;\cap \;p_{mask}$. Further, FP is the region defined by the ground truth that is not segmented through the model formulated as $gt_{mask}\;\cap \;\bar{p}{}_{mask}$. Lastly, TN enfolds the background pixels.

The employed metrics can be formulated as follows:

(5) $$Dice\;=\;\frac{2TP}{\left(2TP\;+\;FP\;+\;FN\right)},$$

(6) $$Sensitivity\;=\;\frac{TP}{\left(TP\;+\;FN\right)},$$

(7) $$Specificity\;=\;\frac{TN}{\left(TN\;+\;FP\right)},$$

The aggregated Jaccard index (AJI) can be defined as:

(8) $$\mathrm{AJI}\;=\;\frac{\sum_{i=1}^{M}\left|{gt_{mask}}_{i}\;\cap \;{p_{mask}^{*}}_{j}\left(i\right)\right|}{\sum_{i=1}^{S}\left|{gt_{mask}}_{i}\;\cup \;{p_{mask}^{*}}_{j}\left(i\right)\right|\;+\;\sum_{S\in \mathrm{Ind}}\;p_{maskS}}$$

where, ${gt_{mask}}_{t}$ is the *i*th ground-truth mask of ICOS cells pixels, $p_{maskS}$ is the predicted cells segmentation mask generated through the model, ${p_{mask}^{*}}_{j}\left(i\right)$ is the connected component from the predicted binary mask that advances the Jaccard index, and Ind is the list of indices of pixels that do not belong to any element in the ground-truth pixels.

### 3.4. Results

In this subsection, we present and discuss the segmentation results of the proposed model with several state-of-the-art methods. We conducted an ablation study including the effect of attention mechanism and the effect of the loss functions.

#### 3.4.1. Effect of Attention Block

Table 1 shows the performance of the proposed model with and without the attention block employed for ICOS positive cells segmentation. We first built the segmentation model without adding the attention block called Baseline, which only combines the plain encoder and decoder structure. The developed baseline model obtained the Dice score of 75.09% and AJI of 59.67%. On seeing the great success of the attention technique on multiple datasets however, we also merged it into our encoder layers. We achieved a better segmentation result than Baseline since the attention block highlighted the most relevant features and ignores the irrelevant ones. Defined kernels must focus on the target nuclei region. Some artifacts however, due to the dye and blurring in certain areas could infer the results. The attention approach enables the filters to focus on capturing the appropriate features and learn the pattern to discriminate between artifacts and nuclei. The proposed model (i.e., baseline + attention block) improved 1% in all the computed metrics. The attention block did not add any extra burden on computational complexity. The proposed model allowed segmentation of the patches with 154 frames per second (FPS) on the current GPU.

#### 3.4.2. Effect of the Loss Function

Table 2 demonstrates the ablation study conducted to measure the effect of a different combination of loss functions on the proposed model. The weighted sum of $L^{\mathrm{BCE}}$ + $L^{\mathrm{Dice}}$ outperformed the segmentation results in all metrics than other individual loss function. This combined loss function achieved the 2–3% better performance in Dice and AJI scores. Additionally, advance the model sensitiveness by 4% compared to only $L^{\mathrm{BCE}}$. The $L^{\mathrm{BCE}}$ obtained lower scores than the proposed combination and generated more falsepositive with higher standard deviation. In turn, $L^{\mathrm{Dice}}$ reduces the pixel-wise false positives leading to better segmentation results. Overall, the combined loss functions provide more generalizability and help converge the model faster with minimal error.

#### 3.4.3. State-of-the-Art Result Comparison

Table 3 presents the result of the proposed model and compared with six state-of-the-art methods. Specifically, we employed the U-Net [24], FCN [25], attention U-Net [26], DeepLabv3+ [27], U-Net++ [28], and Efficient U-Net [20]. The proposed model outperformed the compared methods in segmenting the ICOS positive cells with AJI, sensitivity, and Dice coefficient scores of more than 76%. The second highest U-Net++ achieved the Dice score of 74.93% and AJI with 55.20%, which is 1–5% lower than our proposal. However, U-Net, attention U-Net, and FCN secured similar results in segmenting the positive cells. The proposed model surpasses the DeepLabv3+ with 4.6% and 9.5% on Dice and AJI scores, respectively. We compared the proposed model with previous work performed by [20]. Our model results significantly exceed the earlier work with a margin of 2.5% and 2% in Dice score and AJI. Additionally, we computed each method of computational complexity with corresponding FPS at the inference level. We observed the proposed model shows the lowest trainable parameters of 8.1 M among all and attained the second-best 154 FPS on current GPU. Conclusively, the significant benefit of incorporating the CNN in local level features extraction and Transformer block with attention mechanism allows capturing a global representation of cells structure. The other methods only considered the standard CNN to work well but exhibit some limitations to segmenting the challenging cell structures in the presence of undefined boundaries or artifacts. These issues, at some level addressed by the proposed model, lead to better segmentation results than the rest.

**Figure 3:**
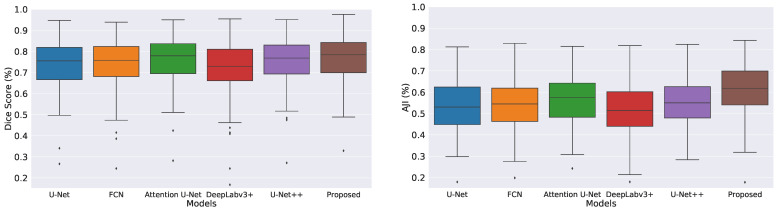
Boxplots of Dice and AJI scores on test dataset. Multiple color boxes highlight the score range of different segmentation methods; the black line inside each box shows the median value, box limits incorporate interquartile ranges Q2 and Q3 (from 25% to 75% of samples), upper and lower whiskers are measured as 1.5× the distance of upper and lower limits of the box, and all values outside the whiskers are assumed outliers.

We measured descriptive statistics of each method’s results using box-plot analysis. Figure 3 shows the box-plots generated with the help of the Dice coefficient and AJI scores obtained by all the evaluated methods. The multiple color boxes highlight the score range of different segmentation methods; the black line inside each box shows the median value, box limits incorporate interquartile spans Q2 and Q3 (from 25% to 75% of samples), upper and lower whiskers are measured as 1.5× the distance of upper and lower limits of the box, and all values outside the whiskers are assumed outliers. We noticed that the proposed model achieved better results than the rest. However, compared methods generated multiple outliers with the lowest Dice and AJI scores.

#### 3.4.4. Qualitative Evaluation

**Figure 4:**
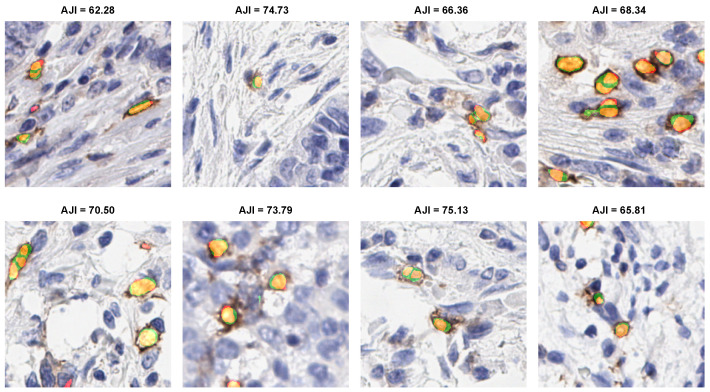
Illustration of proposed model segmentation result. Color maps shows the following description: orange/yellow (true positive), green (false positive), and red (false negative).

The eight qualitative examples obtained by the proposed ICOSeg for ICOS positive cell segmentation are depicted in Figure 4. We used color maps to represent the following description: orange/yellow denotes true positive, green denotes false positive, and red indicate false negative. All the examples presented yielded better results. Visual inspection confirms that ICOSeg generates very few false positives. For any deep neural network, splitting the two connected cells boundaries containing similar dye artifact in certain area is challenging task. The proposed method attempted to capture the majority of the positive cells in patch images.

**Figure 5:**
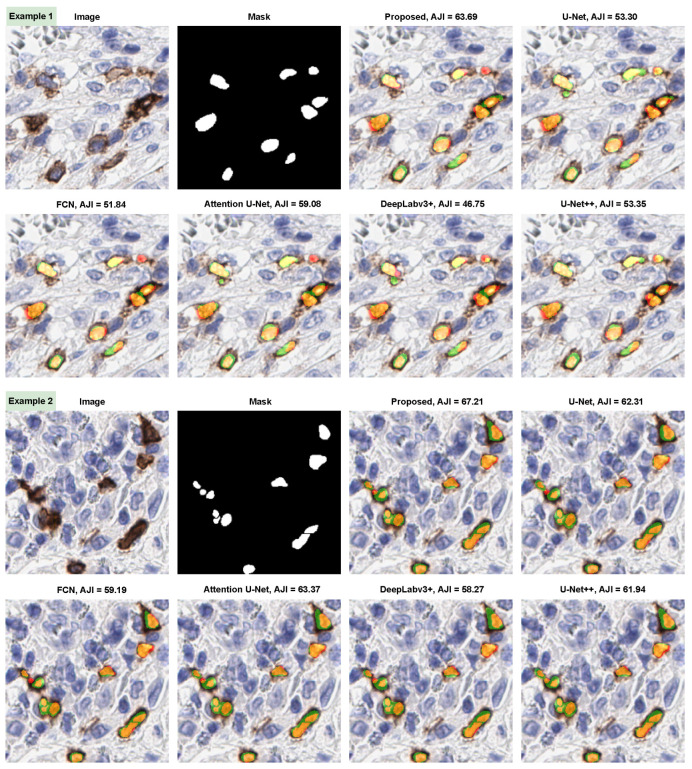
Illustration of proposed model segmentation results compare with five state-of-the-art methods. Color maps shows the following description: orange/yellow (true positive), green (false positive), and red (false negative).

In addition, we provided a comprehensive comparison of ICOSeg with six state-of-the-art methods. Images with the associated mask shown to visualize the cell’s textural patterns and boundaries. Figure 5 represents the two qualitative examples for ICOS positive cell segmentation. This visual finding, combined with quantitative evidence, confirms that the proposed model outperforms the other methods. Figure 5 (i.e., example 1) attempted to segment the small cell boundaries correctly; however, when compared methods performed well but produced more false positives. Similarly, example 2 depicts undefined cell boundaries. The ambiguous cell boundaries were not segmented by any of the six methods, which resulted in it being identified as a single structure. With the combined effect of CNN and transformer, the proposed model allows better feature representation in situations where other models do not show significant improvement.

**Figure 6:**
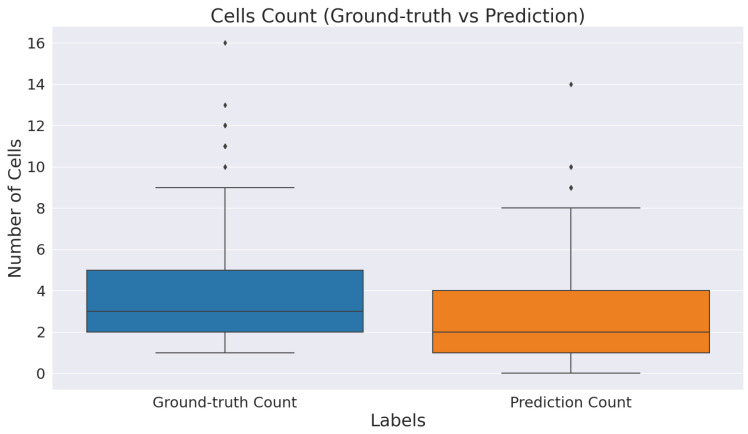
Boxplot of total cell identified by ICOSeg compared to actual cells of the ground-truth.

Figure 6 provides descriptive statistics of the total cell count identified by ICOSeg compared to actual cells of the ground-truth. We count the total number of cells in each patches by finding its contour from image processing operations. There are 642 cells marked by the pathologist in a test set. However, ICOSeg correctly identified the 495 cells with the same set of patches. This plot shows the majority of the patch contains an average of five to seven patches. However, some patches have more than 10 cells represented as outliers in a distribution plot.

### 3.5. Limitation

**Figure 7:**
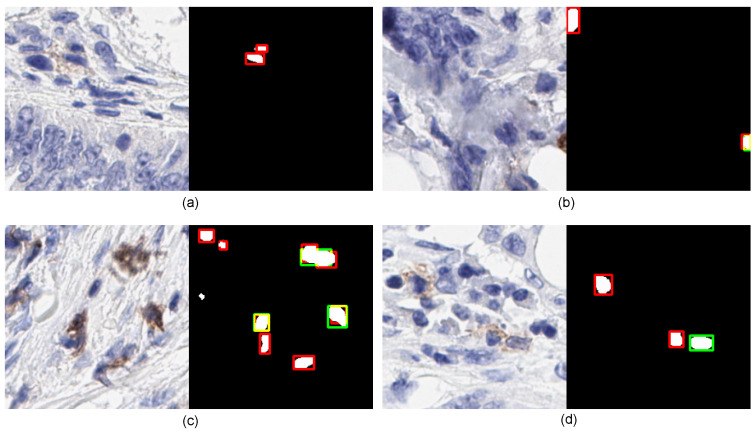
Illustration of four examples shows the limitation of ICOSeg failed to accurately identify and segment the cells boundaries. The red refers to the ground-truth, green corresponds to the ICOSeg prediction and orange/yellow represents the overlap of cell boundary both are common in ground-truth and prediction. The four different examples (**a**–**d**) refers to the positive cells in brown.

Figure 7 shows four qualitative examples that illustrate the limitations of the proposed model on IHC patch images. Example one exhibits an area representing the structure of the normal cell. However, it was marked by a pathologist but failed to recognize by ICOSeg. Next, the second example (see in Figure 7b) has two cells marked in which only one of them correctly identified. The top-left cell presents the structure that more correlates to the normal cells, the proposed model failed to recognize it which was clinically found to be an outlier. In addition, the third and fourth examples (see in Figure 7c,d) show similar characteristics of some positive cells’ ambiguous boundaries that the model failed to provide precise segmentation.

## 4. Conclusions

In this article, we presented an automated lightweight ICOSeg model for ICOS positive cell segmentation. This study is an extension of our previous work that demonstrates higher computational complexity for similar tasks. Our proposed method differs from earlier research findings in several ways. We included the use of Transformers in the encoder for global feature representation with lower computational complexity and attained better segmentation results compared to CNN alone. We used the feature extractor motivated by the MobileViT by adding a channel attention mechanism at the last encoding layer and built the decoder sub-network to reconstruct the features into a binary segmentation map. The proposed model outperformed multiple state-of-the-art segmentation methods with less trainable parameters with higher FPS speed. The lower computational complexity allowed the feasibility of translating the model into a limited resource system. The future work would include extending the potential of our proposed model to multiple protein cell datasets for other cancer types.

## Figures and Tables

**Table 1:** Examining the effect of adopted attention block on segmentation result (mean ± standard deviation) incorporated with Baseline model. The best results are highlighted in bold.

Model	Metrics				Para (M)	FPS
	Dice	AJI	Sensitivity	Specificity		
Baseline	75.09 ± 13.52	59.67 ± 12.90	81.34 ± 15.99	99.54 ± 0.43	8.1	161
**Proposed**	**76.01** ± **13.35**	**60.88** ± **12.85**	**82.45** ± **15.96**	**99.61** ± **0.40**	**8.1**	**154**

**Table 2:** Ablation study of the loss functions. The best results are highlighted in bold.

Model	Metrics				Para (M)	FPS
	Dice	AJI	Sensitivity	Specificity		
$L^{\mathrm{BCE}}$	73.28 ± 15.77	57.41 ± 14.55	78.82 ± 16.61	99.23 ± 0.70	8.1	154
$L^{\mathrm{Dice}}$	74.97 ± 13.83	59.30 ± 13.72	80.65 ± 16.08	99.58 ± 0.41	8.1	154
**$L^{\mathrm{BCE}}$ + $L^{\mathrm{Dice}}$** **(Proposed)**	76.01 ± 13.35	60.88 ± 12.85	82.45 ± 15.96	**99.61** ± **0.40**	**8.1**	**154**

**Table 3:** Segmentation results (mean ± standard deviation) of the proposed model compared with six state-of-the-art methods. The best results are highlighted in bold.

Model	Metrics				Params (M)	FPS
	Dice	AJI	Sensitivity	Specificity		
U-Net [24]	73.66 ± 12.61	53.49 ± 12.75	70.69 ± 15.46	99.71 ± 0.29	34.5	116
FCN [25]	73.79 ± 12.59	53.98 ± 12.57	72.01 ± 15.7	99.68 ± 0.35	135.53	57
Attention U-Net [26]	74.91 ± 13.58	55.66 ± 13.07	74.14 ± 16.05	99.65 ± 0.36	34.87	106
DeepLabv3+ [27]	71.38 ± 14.63	51.21 ± 14.19	66.68 ± 17.78	99.74 ± 0.27	59.33	103
U-Net++ [28]	74.93 ± 12.31	55.20 ± 12.57	72.74 ± 15.40	99.69 ± 0.31	9.61	168
Efficient-UNet [20]	73.44	58.98	81.81	99.30	66.0	83
**Proposed**	**76.01** ± **13.35**	**60.88** ± **12.85**	82.45 ± 15.96	99.61 ± 0.40	**8.1**	**154**

## Data Availability

The images used were derived from tissue samples that are part of the Epi700 colon cancer cohort and were received from the Northern Ireland Biobank. Data availability is subject to an application to the Northern Ireland Biobank with review by the Epi700 committee.
